# Genetic Variations in Crimean-Congo Hemorrhagic Fever Virus: Challenges for Molecular Diagnostic Assays—A Case Report

**DOI:** 10.1155/crdi/4600676

**Published:** 2025-08-05

**Authors:** Mostafa Salehi-Vaziri, Tahmineh Jalai, Mahsa Tavakoli, Laya Farhan Asadi, Seyed Marzieh Sajadi, Tahereh Mohammadi, Sepideh Gerdooei, Sahar Khakifirouz, Farnoosh Arbabi, Arash Ghalyanchi Langeroudi, Mohammad Reza Shirzadi, Mohammad Hassan Pouriayevali

**Affiliations:** ^1^Department of Arboviruses and Viral Hemorrhagic Fevers (National Reference Laboratory), Pasteur Institute of Iran, Tehran, Iran; ^2^Department of Microbiology and Immunology, Faculty of Veterinary Medicine, University of Tehran, Tehran, Iran; ^3^Center for Communicable Diseases Management, Ministry of Health and Medical Education, Tehran, Iran

**Keywords:** arbovirus, Crimean–Congo hemorrhagic fever, emerging infectious diseases, Iran, laboratory diagnosis, tick-borne viral diseases

## Abstract

**Background:** Crimean–Congo hemorrhagic fever (CCHF) is a tick-borne viral disease with a high mortality rate which is endemic in Iran. Laboratory diagnosis of CCHF is routinely conducted using PCR and IgM ELISA tests. However, nucleotide variations within CCHF virus (CCHFV) may lead to false-negative PCR results.

**Case Presentation:** A 51-year-old patient suspected to have CCHF was tested for CCHFV infection using two different molecular assays. The results were discrepant, as our homemade SYBR green-based real-time PCR yielded a strong positive result, while the RealStar CCHFV RT-PCR Kit returned a negative result. Due to the discrepancies in the real-time PCR tests, a homemade conventional RT-PCR method was performed, resulting in a positive result similar to the SYBR green-based real-time PCR assay. Partial sequencing of S segment of viral genome and phylogenetic analysis revealed that the strain clustered with a strain from Africa-3 genotype, which was isolated in Namibia in 1987. CCHF antigen and IgM ELISA also confirmed the CCHFV infection in this case.

**Conclusions:** This report underlines the requirement of CCHFV genomic surveillance to update the molecular diagnostic assays. Moreover, the circulation of an African CCHFV strain in Iran supports previous data suggesting that Iran harbors the greatest CCHFV genetic diversity among endemic countries. Discrepancies in PCR results, likely due to this diversity, may hinder timely diagnosis and subsequently affect patient management and treatment measures.

## 1. Background

Crimean–Congo hemorrhagic fever (CCHF) is one of the most important tick-borne viral diseases, posing a considerable health threat due to its high mortality rate (up to 50%) and the absence of specific antiviral therapy and approved vaccines [1]. Endemic in over 31 countries across Africa, Europe, and Asia, the disease puts approximately 3 billion people at risk, with 10–15 thousand annual cases and 500 resulting in fatalities. Iran is one of the CCHF endemic countries in the Middle East and reports at least 50 laboratory confirmed CCHF cases annually (https://www.who.int/health-topics/crimean-congo-haemorrhagic-fever).

The causative agent, an enveloped virus with a negative-sense RNA genome, belongs to the *Orthonairovirus* genus and the Nairoviridae family [2]. The CCHF virus (CCHFV) genome comprises three fragments (S, M, and L), encoding the virus's nucleoprotein, surface glycoproteins, and viral RNA-dependent RNA polymerase enzyme (RdRp), respectively.

CCHFV exhibits genetic diversity with seven genotypes (Asia 1 and 2, Africa 1, 2, and 3, Europe 1 and 2). In terms of evolution, the S segment is the most conserved, followed by the L and M [3] segments. Recently, the Europe 2 genotype of CCHFV also known as AP-92 like or Genotype VI, was re-classified as a distinct virus, Aigai virus (AIGV) by the International Committee on Taxonomy of Viruses (ICTV) [4].

Routine laboratory diagnosis of CCHF relies on PCR and IgM ELISA tests, with PCR methods typically targeting the S segment due to its genetic stability [5]. However, nucleotide variations even in S segment may lead to false-negative PCR results, prompting the recommendation to complement molecular methods with serological tests. It is reasonable to use at least two targets for virus genome identification. The reported case highlights an instance of a false-negative PCR result in a CCHF patient, emphasizing the importance of a comprehensive diagnostic approach.

## 2. Case Presentation

On November 11, 2023, a 51-year-old male farmer residing in a village in Shadegan city, Khuzestan province (southwest Iran), sought medical attention at Ahvaz Hospital due to abrupt onset of weakness, lethargy, myalgia, abdominal pain, followed by nausea, vomiting, hematemesis, and hematuria. Subsequent investigations, considering clinical symptoms and epidemiological factors such as contact with bodily fluids and tissues from slaughtered livestock and the presence of ticks at the residence, along with observed decreases in hemoglobin and thrombocytopenia in the initial test results, raised suspicions of CCHF. Therefore, a serum sample from the patient was collected on November 15, 2023, and referred to the Department of Arboviruses and Viral Hemorrhagic Fevers (National Reference Laboratory) at the Pasteur Institute of Iran for laboratory diagnosis of CCHF.

The serum sample underwent evaluation for the presence of the CCHFV genome, as well as IgG/IgM antibodies (Table 1). In the first step, a homemade SYBR green-based real-time RT-PCR and a commercial TaqMan real-time PCR kit (The RealStar CCHFV RT-PCR Kit (Altona Diagnostics, Germany)) were employed for viral RNA identification. RNA extraction was carried out using Nucleic Acid Extraction Kit (Zybio, China) by the automated nucleic acid isolation system EXM3000 (Zybio, China) according to manufacturer's instructions. The homemade SYBR green-based real-time RT-PCR was performed using 4X CAPITAL 1-Step qRT-PCR Green Master Mix (Biotech rabbit, Germany) following manufacturer's instructions. Briefly, each 20 μL reaction contained 5 μL of 4x Master Mix, 0.2 μL of each primer (0.05 μM final concentration each), 1 μL RNase inhibitor (Biotech rabbit, Germany) 8.6 μL of nuclease-free water and 5 μL of extracted RNA. The cycling conditions were as follows: one cycle of reverse transcription at 50°C for 10 min, one cycle of initial denaturation at 95°C for 3 min, 45 cycles of 95°C for 10 s and 60°C for 30 s and finally a melting curve ranging from 65°C to 99°C. The commercial TaqMan real-time PCR was performed according to the manufacturer's instructions. Both real-time PCR assays were done using a Rotor-Gene Q system (Qiagen, Germany).

For IgM/IgG detection, the VectoCrimean-CHF-IgM and VectoCrimean-CHF-IgG (Vector Best, Russia) were utilized according to the manufacturer's instructions.

**Figure 1:**
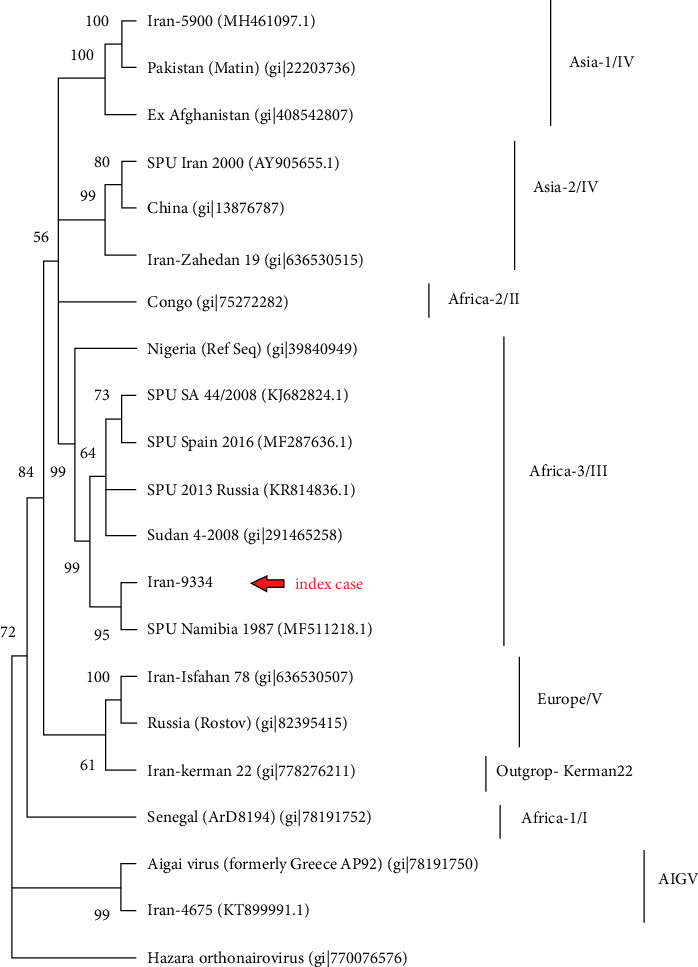
Neighbor-joining phylogenetic tree with bootstrap 5000 and Kimura 2-parameter substitution model indicating that the strain Iran 9334 belongs to CCHFV genotype Africa 3.

Notably, the SYBR green-based real-time PCR test yielded a positive result with a low cycle threshold (ct = 19), indicating a high viral load. Conversely, The RealStar CCHFV RT-PCR Kit (Altona Diagnostics, Germany) returned a negative result. The IgM ELISA result was borderline and the IgG ELISA tested negative. Due to the discrepancies in the molecular test results, a homemade conventional RT-PCR method using F2 and R3 primers was applied [6], resulting in a positive result similar to the SYBR green-based real-time PCR assay. For phylogenetic analysis S segment sequencing was performed by the Sanger sequencing method with an ABI 3500 sequencer. The S segment was amplified and sequenced using two sets of specific sequencing primers (F Set 3: TGGCAAAATGGAAAACAA and R3: GACAAATTCCCTACACCA). Reads were assembled against the Iran-5900 reference, generating a 622 nt consensus sequence (from nucleotide 32 to 654 based on Iran-5900 GenBank: MH461097.1). This consensus sequence was aligned with 20 sequences retrieved from GenBank using Clustal W in MEGA 11 software. The selected sequences covered various genotypes of CCHFV, AIGV (formerly CCHFV Europe 2), and one sequence of Hazara virus serving as the root for an unrooted phylogenetic tree. The evolutionary history was determined using the Neighbor-Joining method. The resulting tree displays the best configuration, with the percentage of grouped taxa appearing together in the bootstrap test (5000 replicates) indicated by each branch (values under 55% are not shown). Evolutionary distances were calculated using the Kimura 2-parameter method, measured in base substitutions per site (Figure 1). Interestingly, our sequence clustered with a strain from Africa-3 genotype which was isolated in Namibia in 1987. The sequence was submitted to GenBank with the accession number PP496956.

Subsequently, an antigen ELISA assay was carried out using VectoCrimean-CHF-antigen kit (Vector Best, Russia), which showed a positive result, confirming the initial findings. In order to observe seroconversion, a second serum sample was collected from the patient on November 25, 2023, and analyzed for IgM and IgG. The results showed a strong positive IgM and borderline IgG, indicating seroconversion. Table 1 provides an overview of all laboratory tests conducted on the two prepared samples.

According to the national protocol for the treatment of suspected CCHF cases in Iran, supportive treatment and ribavirin administration were performed and finally the patient was discharged on November 27 [7].

## 3. Discussion and Conclusions

Here, we report an Iranian CCHF patients infected with an uncommon strain of CCHFV belonging to Africa 3 genotype which showed discrepant results in molecular assays. Among three molecular assays targeting the S segment of the CCHFV genome, our in-house assays including a SYBR green-based real-time RT-PCR and a conventional RT-PCR yielded positive results. In contrast, no amplification signal was detected using the commercial CCHF real-time PCR (Altona), highlighting potential limitations in the assay sensitivity or primer-target mismatches due to viral genetic diversity. There are several lines of evidence that CCHFV is one of the most diverse viruses in terms of genetic variation. Genetic drift due to accumulation of point mutations related to the activity of the error-prone viral RdRp, genetic recombination and genetic reassortment together contribute to the viral genome sequence diversity of CCHFV [3]. Although the S segment of CCHFV genome, the most conserved region compared to L and M segments, is routinely used as the target for nucleic acid amplification testing [5], the sensitivity of these assays may still be hampered by the broad genetic diversity of the virus. Usually, molecular assays for CCHFV are being designed using a limited number of strains from specific geographical areas and hence these tests may suffer from low sensitivity to identify nonendemic strains from other genotypes [8]. For instance, Vanhomwegen et al. indicated that the RealStar CCHFV RT-PCR Kit (Altona-Diagnostics, Germany) was less sensitive for Turkish and Albanian samples [9]. In an international external quality assessment program (EQAP) of molecular diagnosis of CCHFV in which 44 laboratories from 29 different countries worldwide participated, optimal performance, and acceptable performance were observed only in 38% and 19% of data sets, respectively [10]. These findings altogether emphasize the need to improve CCHFV molecular diagnostic assays. In this regard, several strategies can be adopted: (1) designing molecular assays against as many as CCHFV strains from all endemic countries, (2) designing molecular assays targeting at least two regions of the S segment of CCHFV genome to minimize the adverse effects of genetic diversity, and (3) performing molecular assays in combination of serological tests such as IgM ELISA to improve the sensitivity of CCHFV diagnostic algorithm.

In the National Reference Laboratory of Arboviruses and viral hemorrhagic fevers at Pasteur Institute of Iran, we use two different real-time PCR assays with different primer binding sites on S segment, in combination of IgM ELISA for detection of acute infection of CCHFV in suspected patients [11].

Owing to ongoing genetic diversity of CCHFV, circulating strains even in one endemic country can undergo significant changes over time and therefore, regular monitoring of CCHFV strains in endemic countries should be implemented (preferentially using next-generation sequencing [NGS]) to evaluate the efficacy of molecular assays and updated them if necessary [5]. Interestingly, in the study by Vanhomwegen et al. [9] which was conducted in 2012, the RealStar CCHFV RT-PCR Kit (Altona-Diagnostics, Germany) showed a sensitivity rate of 100% to detect CCHFV strains from Iran, while this kit failed to detect CCHFV infection in our case. Monitoring the CCHFV strains and updating the molecular tests is of utmost importance in countries like Iran, where a notable rate of CCHFV genetic diversity has been reported. Genomic surveillance and phylogenetic analysis is a powerful tool for characterizing the genetic diversity of CCHFV, enabling identification of distinct viral lineages and their geographical origins. These tools are crucial for informing surveillance efforts, updating diagnostic tools, and anticipating potential shifts in viral epidemiology that may affect public health responses. Since the first identification of human CCHFV infection in 2000 in Iran, at least 5 genotypes (Africa-3, Asia-1, Asia-2, Europe-1, and Europe 2 [aka AIGV]) of CCHFV have been documented in the country [3, 6].

The phylogenetic analysis revealed that our case was infected with a strain belonging to the Africa-3 genotypes and closely related to strains from Namibia. Although the circulation of CCHFV Africa-3 genotype has been documented in Iran, it dated back in 1980 [12] and this genotype has not been reported in Iran from that moment until now. Re-emergence of a CCHFV strain from Africa-3 genotypes in Iran can be explained by the introduction of the virus to Iran via tick-infested migratory birds, a documented route of CCHFV spread across countries [12–14]. To the best of our knowledge, there has been no study to investigate the CCHFV infection in ticks imported via migratory birds or livestock trade to Iran. Another hypothesis could be that CCHFV Africa-3 genotype has been circulating in Iran but at a lower prevalence and under the radar. The second justification is highly unlikely because genomic surveillance of CCHFV is conducted annually in Iran.

In conclusion, this case report highlights the critical need for ongoing genomic surveillance of CCHFV to determine the circulating strains and ensure the sensitivity of molecular diagnostic assays. Additionally, the detection of an African-3 genotype strain previously unreported in Iran since 1980 underscores the country's extensive CCHFV genetic diversity and suggests potential reintroduction routes, such as migratory birds. These findings emphasize the importance of regulatory updating diagnostic tools to account for the evolving genetic landscape of CCHFV, particularly in endemic regions like Iran, where multiple viral genotypes co-circulating and false-negative results can hamper case finding, patient management, infection prevention control measures, and contact tracing.

## Figures and Tables

**Table 1:** CCHFV infection diagnostic tests performed on two serum samples.

Sample	Date of sampling	Molecular tests				ELISA tests		
		SYBR green-based real-time PCR	RealStar CCHFV RT-PCR kit	Conventional RT-PCR [6]	CCHFV genotype	IgM (OD)	IgG (OD)	Antigen (OD)
Sample 1	15 Nov 2023	Positive (ct = 19.5, melt = 83.2)	Negative	Positive	Africa-3/III	Borderline (0.22)	Negative (−)	Positive (1.1)
Sample 2	25 Nov 2023	ND	ND	ND	—	Positive (OVFL)	Borderline (0.24)	Negative (−)

## Data Availability

The data that support the findings of this study are available from the corresponding author upon reasonable request.

## Nomenclature

AIGV: Aigai virus
CCHF: Crimean–Congo hemorrhagic fever
CCHFV: Crimean–Congo hemorrhagic fever virus
ELISA: Enzyme-linked immunosorbent assay
ICTV: International Committee on Taxonomy of Viruses
RdRp: RNA-dependent RNA polymerase enzyme
RT-PCR: Reverse transcription polymerase chain reaction
